# Global Spread of Vancomycin-resistant *Enterococcus faecium* from Distinct Nosocomial Genetic Complex

**DOI:** 10.3201/eid1106.041204

**Published:** 2005-06

**Authors:** Rob J.L. Willems, Janetta Top, Marga van Santen, D. Ashley Robinson, Teresa M. Coque, Fernando Baquero, Hajo Grundmann, Marc J.M. Bonten

**Affiliations:** * University Medical Center Utrecht, Utrecht, the Netherlands;; † National Institute for Public Health and the Environment, Bilthoven, the Netherlands,; ‡ New York Medical College, Valhalla, New York, USA;; § Hospital Ramon y Cajal, Madrid, Spain

**Keywords:** molecular epidemiology, Enterococcus faecium, Multilocus Sequence Typing, Population structure, Genetic evolution, VRE, vancomycin-resistance

## Abstract

Vancomycin-resistant enterococci (VRE) have caused hospital outbreaks worldwide, and the vancomycin-resistance gene (*vanA*) has crossed genus boundaries to methicillin-resistant *Staphylococcus aureus*. Spread of VRE, therefore, represents an immediate threat for patient care and creates a reservoir of mobile resistance genes for other, more virulent pathogens. Evolutionary genetics, population structure, and geographic distribution of 411 VRE and vancomycin-susceptible *Enterococcus faecium* isolates, recovered from human and nonhuman sources and community and hospital reservoirs in 5 continents, identified a genetic lineage of *E. faecium* (complex-17) that has spread globally. This lineage is characterized by 1) ampicillin resistance, 2) a pathogenicity island, and 3) an association with hospital outbreaks. Complex-17 is an example of cumulative evolutionary processes that improved the relative fitness of bacteria in hospital environments. Preventing further spread of this epidemic *E. faecium* subpopulation is critical, and efforts should focus on the early disclosure of ampicillin-resistant complex-17 strains.

The emergence of vancomycin-resistant enterococci (VRE) followed a worst-case scenario for nosocomial pathogens: the first VRE isolates that harbored the *vanA* transposon were identified in 1987 in Europe (1,2), and within 10 years VRE represented >25% of enterococci associated with bloodstream infections in hospitalized patients in the United States (3).

Enterococci are normal inhabitants of the gastrointestinal tract of humans and animals. Two species cause most enterococcal infections, *Enterococcus faecalis* and *E. faecium*. The relative importance of *E. faecium* as a pathogen has increased with the occurrence of high-level resistance to multiple antimicrobial drugs, such as ampicillin and vancomycin (4). The rapid increase of vancomycin resistance compromises physicians' ability to treat infections caused by many of these strains because often no other antimicrobial drugs are available. The epidemiology of VRE infection differs between Europe and the United States. In Europe, VRE are frequently isolated from farm animals, which have been associated with the abundant use of avoparcin as a growth promoter in the agricultural industry, until it was banned in 1997 (5). The reported prevalence of VRE in hospitals has been low, but increasing rates (>10%) in stool and clinical samples were reported recently (6–9). In the United States, avoparcin was never approved for use in agriculture, and neither were any other glycopeptides; consequently, VRE have not been found in animals or healthy persons. However, nosocomial VRE infection and transmission have occurred much more frequently in the United States. Recent reports have documented, in hospitalized patients, horizontal transfer of the *vanA* gene from vancomycin-resistant *E. faecalis* to methicillin-resistant *Staphylococcus aureus* (MRSA), creating MRSA with high-level resistance to vancomycin (10–13). Nosocomial spread of VRE may therefore create a reservoir of mobile resistance genes for other, more virulent, nosocomial pathogens. Without extensive control measures, large-scale emergence of vancomycin-resistant *S. aureus* (VRSA) may be the next stage in the global crisis of antimicrobial resistance.

The existence of VRE in different ecologic niches complicates the understanding of its epidemiology. Although previous molecular epidemiologic studies on limited numbers of strains suggested host specificity and overrepresentation of certain clones in hospital outbreaks (14,15), these studies did not elucidate the patterns of evolutionary descent among VRE. We determined the population structure of 411 VRE and vancomycin-susceptible *E. faecium* (VSE) isolates by using multilocus sequence typing (MLST), explored the evolutionary origin of epidemic isolates associated with documented hospital outbreaks and other isolates, and assessed the association with ampicillin resistance and the presence of a recently discovered putative pathogenicity island (PAI) in *E. faecium* (16).

## Materials and Methods

The strain collection included 5 categories of VRE and VSE: 1) 96 animal surveillance (bison, calves, cats, dogs, ostriches, poultry, pigs, rodents) isolates (43 VRE, 53 VSE) from 7 countries in Africa and Europe; 2) 57 epidemiologically unrelated community surveillance isolates (20 VRE, 37 VSE) from nonhospitalized persons from 7 countries in Australia and Europe; 3) 64 epidemiologically unrelated surveillance (fecal) isolates (45 VRE, 19 VSE) from hospitalized patients not linked to hospital outbreaks from 9 countries in Australia, Europe, and North and South America; 4) 162 epidemiologically unrelated hospital isolates (43 VRE, 118 VSE, 1 not determined) from clinical specimens (blood, pus, and urine) from 17 countries in Africa, Australia, Europe, and North and South America; and 5) 1 strain from each of 32 different documented hospital outbreaks (28 VRE, 4 VSE) in 10 countries in Australia, Europe, and North and South America (W. Grubb and D. Jonas, pers. comm.; 15,17–23).

We determined vancomycin susceptibilities for 410 isolates and ampicillin susceptibilities for 381 isolates by using standard agar dilution methods according to NCCLS guidelines. Isolates with MIC ≥16 μg/mL for ampicillin and ≥8 μg/mL for vancomycin were considered to be resistant. In total, 394 strains were screened for the *esp* gene with primer sets and amplification conditions described previously (24). Independent and combined effects of virulence and resistance markers on the abundance of complex-17 were estimated by using multiple logistic regression analysis (Stata 7.0, StataCorp LP, College Station, TX, USA).

MLST was carried out with a standard set of primers that amplify the 7 genes included in the *E. faecium* MLST scheme (14). Information on these loci, the latest set of primers, amplification conditions, and details of all isolates are available on the MLST Web site (http://efaecium.mlst.net).

The eBURST program was used to assess the genetic relationships of genotypes, to assign isolates to genetic complexes, and to study patterns of evolutionary descent of isolates within a complex (25). Complexes were identified by using the stringent (6/7 shared alleles) group definition with 1,000 bootstrap replicates. The BLAND program was used to examine the relationship between pairwise allelic differences and nucleotide sequence differences (26). If genetic diversity in *E. faecium* is mainly the result of accumulated point mutations, then recently diverged strains will have a high level of similarity in both their allelic profiles as well as in the nucleotide sequence of the nonidentical alleles, which results in a positive correlation between the number of nucleotide differences in nonidentical alleles and the number of allelic differences. However, such a trend will be absent when recombination plays an important role in generating the genetic diversity, since nonidentical alleles of closely related isolates can differ at multiple nucleotide sites.

To assess the effect of recombination on the population structure of *E. faecium* in more detail, the topologies of the 7 MLST gene trees were compared by using the Shimodaira-Hasegawa test (27). Briefly, maximum likelihood trees for each MLST gene were obtained under a general time-reversible model, with a proportion of invariant sites and rate heterogeneity among sites assuming a discrete gamma distribution with 8 categories (GTR+I+Γ model). PAUP* 4.0b10 was used to obtain the maximum likelihood trees by using a neighbor-joining starting tree followed by tree-bisection reconnection branch swapping (28). For a given gene, the Shimodaira-Hasegawa test compares the difference in log likelihoods of competing tree topologies. A null distribution of differences in log likelihoods was obtained by 1,000 replicates of nonparametric bootstrapping of reestimated log likelihoods. We conducted 107 Shimodaira-Hasegawa tests for each MLST gene by comparing the 7 MLST gene trees and 100 random trees separately generated for each of the MLST genes. In a clonal population, the different MLST housekeeping genes have similar tree topologies, but with recombination, the different genes may have different tree topologies that may fit random trees better.

Associations between ampicillin resistance, presence of a novel putative *E. faecium* PAI, and genetic clustering in complex-17 were described by linear logistic regression models: log odds = b_0_ + b_1_x_1_ + b_2_x_2_ + b_3_x_3_ + … + b_i_x_i_. Log odds denotes the natural logarithm of the proportion of samples from an epidemiologic group belonging to complex-17, b_i_ denotes the parameter estimated by maximum likelihood methods and x_i_ the level of exposure, e.g., 0 and 1 for ampicillin resistance, vancomycin resistance, and the presence of PAI and 0–4 for the epidemiologic source of isolates: animal surveillance, community surveillance, hospital surveillance, clinical sample, and hospital outbreak, respectively.

## Results

### Identification of Clonal Lineages

**Figure 1:**
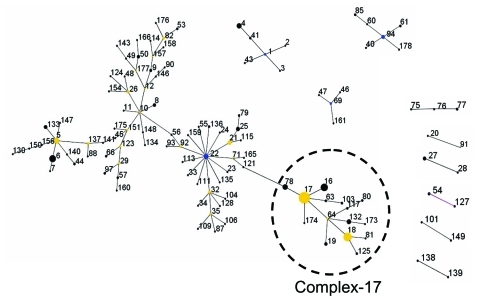
Clustering of 175 sequence types representing 411 isolates with eBURST (25). This algorithm identifies the founder of a complex or genogroup of related sequence types (ST) and subsequent patterns of evolutionary descent. The primary founder, indicated in blue, of a complex is defined as the ST with the largest number of single locus variants (SLVs). Larger complexes may contain secondary founders of additional lineages that have a number of SLVs of their own. These secondary founders are indicated in yellow. Numbers correspond to ST numbers. The area of each circle corresponds to the number of isolates of the ST. All complexes (major and minor) are shown. In addition, 57 STs did not group into any of the complexes and were considered singletons (STs 13, 15, 30, 31, 36, 37, 38, 39, 42, 51, 52, 58, 59, 62, 65, 67, 68, 70, 72, 73, 74, 83, 84, 86, 89, 95, 96, 98, 99, 100, 102, 105, 107, 108, 110, 112, 114, 116, 118, 126, 129, 131, 142, 144, 145, 152, 153, 155, 162, 163, 164, 167, 168, 169, 170, 171, 172). The "epidemic" genetic complex-17 derived from secondary founder ST-17 is indicated. A measurement of statistical confidence in each of the assigned primary founders is made by a bootstrap resampling procedure (25).The predicted primary founders of the complexes 22, 94, 1, and 69 have a bootstrap value of 73%, 84%, 85%, and 59%, respectively.

MLST of 411 *E. faecium* isolates resulted in 175 different sequence types (ST). Clustering these types with the eBURST algorithm (25) showed 1 large complex of genetically related types. ST-22 was the primary founder; 3 minor complexes had ST-1, -69, and -94 as primary founders; 6 complexes had only 2 or 3 STs; and 57 singletons were not linked to the aforementioned complexes (Figure 1). Within complex-22, ST-17 represents an important secondary founder of a distinct branch designated complex-17.

### Selective Advantage of the Successful Hospital-adapted Complex-17

In all, 142 of 411 isolates belonged to complex-17, with a gradual increase in proportion among animal isolates (1/96), human community isolates (3/57), human hospital surveillance isolates (15/64), and human clinical isolates (95/162), to hospital-outbreak isolates (28/32) (Table 1). Ampicillin resistance, presence of the *E. faecium* PAI (16), and genetic clustering in complex-17 were strongly associated (Table 2).

**Table 1:** Frequency of ampicillin and glycopeptide resistance, the presence of the pathogenicity island (PAI), and log odds of all complex-17 and non–complex-17

Epidemiologic source	Genetic and phenotypic features*														
	Complex-17		Other†		Complex-17		Other†		Complex-17		Other†		Complex-17‡	Other†‡	Log odds§
	AmR	AmS	AmR	AmS	PAI+	PAI–	PAI+	PAI–	VanR	VanS	VanR	VanS			
Animal surveillance (n = 96), %	1	0	2	93	0	1	0	94	0	1	43	52	1 [1]	95 [99]	–4.55
Community surveillance (n = 57), %	0	0	1	46	3	0	0	47	3	0	17	37	3 [5]	54 [95]	–2.89
Hospital surveillance (n = 64), %	14	0	7	40	7	8	0	49	13	2	32	17	15 [23]	49 [77]	–1.18
Clinical (n = 162), %	85	2	13	47	47	47	4	57	21	73	22	45	95 [59]	67 [51	0.35
Hospital outbreak (n = 32), %	26	0	3	1	20	6	3	1	24	4	4	0	28 [88]	4 [12]	1.95

*Ampicillin resistant (AmR) or susceptible (AmS) not determined in 30 isolates, PAI present (PAI+) or absent (PA–) not determined in 17 isolates, vancomycin resistant (VanR) or susceptible (VanS) not determined in 1 isolate. †Not belonging to complex-17. ‡Numbers in brackets refer to the percentage of isolates that belong to the complex. §The natural logarithm of the proportion of samples from an epidemiologic source belonging to complex-17.

**Table 2:** Parameter estimates by using a logistic regression model*

Regression lines	Parameter estimates					
b_0_	b_amp_	b_PAI_	b_gly_	b_epi_	p value
Complex-17 crude	–4.44	0	0	0	1.6	0.000
Corrected for amp	–6.61	5.38	0	0	1	0.000
Corrected for PAI	–6.29	5.08	1.06	0	0.84	0.038
Corrected for gly	–6.07	5.12	1.01	–0.45	0.83	0.316

*Amp, ampicillin resistance; PAI, presence of the *Enterococcus faecium* pathogenicity island; gly, glycopeptide resistance; epi, epidemiologic source (animal surveillance, community surveillance, hospital surveillance, clinical sample, hospital outbreak).

**Figure 2:**
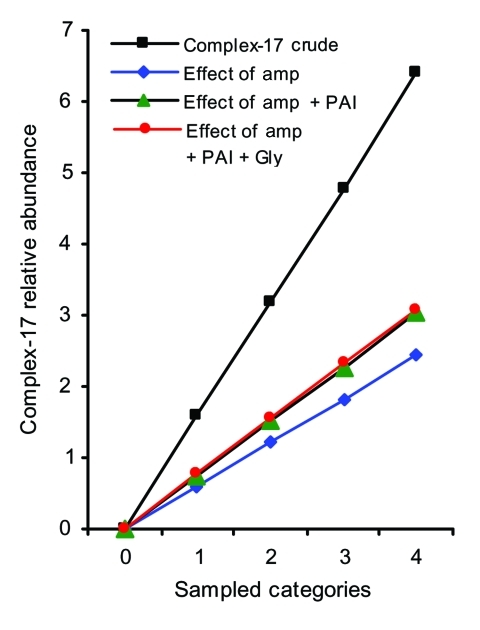
Relative abundance of complex-17 in various sampled categories and proportion increase explained by combined effect of 3 parameters. 0, animal surveillance samples; 1, human community surveillance samples; 2, human hospitalized patient samples; 3, human clinical samples; 4, hospital outbreak samples; amp, ampicillin resistance; PAI, pathogenicity island; gly, glycopeptide resistance.

When controlling for individual and combined effects of ampicillin resistance, presence of PAI, and vancomycin resistance, we can show that 1) the loglinear assumption holds for all effect parameters, and linear models describe the observed frequencies without substantial loss of goodness of fit; 2) individual genetic markers exert an independent and multiplicative effect; and 3) all genetic markers combined explain ≈48% of the category-specific abundance of complex-17 (Figure 2). The effect of vancomycin resistance did not increase the explanatory value of the model, owing to the fact that determinants for vancomycin resistance could be found in equal proportions within and outside of complex-17, likely a result of widespread horizontal transfer of *vanA* (Table 1). This finding suggests that the epidemiologic success of descendants of ST-17 that results in clinical infections and hospital epidemics was at least partly related to antimicrobial resistance and the presence of putative virulence genes. The fact that 126 of 128 isolates of complex-17 were resistant to ampicillin and only 77 of 139 isolates of complex-17 contain PAI (Table 1) suggests that *E. faecium* acquired ampicillin resistance first, which resulted in a selective advantage in hospitals, followed by the acquisition of PAI, which further facilitated transmission.

### Estimates of Recombination

**Figure 3:**
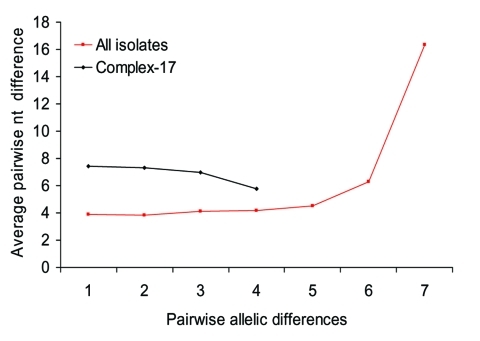
Sequence diversity versus allelic diversity. The average number of nucleotide (nt) differences in nonidentical alleles for all pairwise comparisons of the 178 *Enterococcus faecium* sequence types (STs), and the 15 STs belonging to complex-17 was calculated separately for allelic profiles that differ in 1–7 alleles. This computation shows no positive correlation between the number of nucleotide differences and allelic differences, which suggests that recombination has played an important role in the genetic diversification in *E. faecium*, including the STs that constitute complex-17.

To assess the effect of recombination on the population structure of *E. faecium*, we estimated whether single locus variants (SLVs) from the presumed founders of complex-1, -17, -22, -69, and -94 have arisen by point mutations or by recombination (Table 3). Of all allelic differences between ancestor-SLV pairs (n = 30), 22 (4 in complex-17) included >1 nucleotide, were found in multiple clonal complexes, and thus were most likely a result of recombination. Eight allelic differences (2 in complex-17) included only a single nucleotide change, were unique within the dataset, and thus were most likely a result of mutation. Therefore, most alleles of SLVs in complex-17 and in the other complexes have arisen by recombination in the initial stages of diversification rather than by de novo point mutation. An important role for recombination in genetic diversification in *E. faecium* was confirmed by the lack of a positive trend between the number of nucleotide differences in nonidentical alleles and the number of allelic differences (Figure 3) (26). The finding of high average numbers (≥4) of nucleotide differences in the nonidentical alleles of SLVs in the total *E. faecium* population as well as in complex-17 also points towards frequent recombination.

**Table 3:** Variant alleles of single locus variants (SLVs) within 5 genetic complexes*

ST of ancestor	ST of SLV	Variant locus	Ancestral allele	SLV allele	No. nucleotide differences (amino acid change)
17	64	*atpA*	1	7	4
17	117	*atpA*	1	9	20
17	78	*atpA*	1	15	22
17	16	*ddl*	1	2	1
17	63	*purK*	1	21	1 (C-Y)†
17	174	*purK*	1	29	1†
22	32	*atpA*	2	3	2
22	21	*atpA*	2	9	18
22	92	*atpA*	2	5	19
22	71	*atpA*	2	15	20
22	135	*atpA*	2	27	18
22	159	*atpA*	2	30	1†
22	113	*atpA*	2	26	19
22	55	*ddl*	3	1	6
22	111	*gdh*	1	6	1
22	24	*purK*	2	7	3
22	136	*purK*	2	26	1 (H-Y)†
22	33	*pstS*	1	5	1 (L-V)†
22	23	*adk*	1	7	1†
1	43	*atpA*	8	3	16
1	41	*purK*	7	3	4
1	2	*gyd*	1	9	1 (Y-H)†
1	3	*pstS*	1	12	1 (Y-N)†
94	40	*atpA*	13	10	3
94	60	*gyd*	6	11	1
94	61	*pstS*	10	17	4
94	178	*pstS*	10	27	2
69	46	*atpA*	9	5	1
69	161	*atpA*	9	3	16
69	47	*adk*	6	5	4

*Genetic complexes 1, 17, 22, 69, and 94 were included in this analysis. ST, sequence type; C, cysteine; Y, tyrosine; H, histidine; L, leucine; V, valine; N, asparagine. †Single nucleotide changes that are unique in the dataset and thus are due to mutation.

The degree of phylogenetic congruence between the 7 MLST genes was examined in a set of 24 diverse STs. These 24 STs were separated from each other by a linkage distance of >0.4 on a UPGMA (unweighted pair-group method with arithmetic mean) tree constructed from the pairwise comparisons of their allelic profiles (data not shown) and included the primary founders of CC1 (ST1), CC22 (ST22), CC69 (ST69), and CC94 (ST94); secondary founders of important subgroups complex-5 (ST5) and complex-17 (ST17); 1 ST (ST76) belonging to a small complex of 3 STs; and 17 singletons (STs 15, 38, 39, 54, 67, 74, 83, 84, 89, 96, 98, 99, 101, 107, 118, 142, 163). The results of the congruence analysis presented in Table 4 show that 25 (60%) of 42 of the pairwise comparisons of the 7 MLST loci were incongruent. Of the 7 genes, *atpA* is the most incongruent. This analysis confirms that recombination played a substantial role in the evolution of *E. faecium*.

**Table 4:** Summary of gene congruence analysis

Gene	No. incongruence genes by SH test*	Random trees†
*adk*	1 (*atpA*)	8 (*atpA*)
*atpA*	6 (*adk*, *ddl*, *gdh*, *gyd*, *pstS*, *purK*)	76 (*adk*)
*ddl*	6 (*adk*, *atpA*, *gdh*, *gyd*, *pstS*, *purK*)	8 (*atpA*)
*gdh*	1 (*atpA*)	1 (*atpA*)
*gyd*	2 (*adk*, *atpA*)	0 (*atpA*)
*pstS*	6 (*adk*, *atpA*, *ddl*, *gdh*, *gyd*, *purK*)	3 (*atpA*)
*purK*	3 (*atpA*, *adk*, *gyd*)	1 (*atpA*)

*Number of incongruent genes at the p<0.05 level based on a Shimodaira-Hasegawa (SH) test of tree topologies. The incongruent genes are in parentheses. †Number of random tree topologies out of 100 random trees that are better fit to the gene tree from the most incongruent multilocus sequence typing (MLST) gene. The most incongruent MLST gene is given in parentheses.

## Discussion

Nosocomial VRE, which rapidly emerged in the United States in the 1990s after their initial discovery in Europe, are found in increasing rates in hospitals in Europe, Asia, and South America (5–7,9,23,29,30). The data presented in this study show that most of these hospital-derived VRE are part of a single clonal lineage. This lineage, designated complex-17 after its presumed founder ST-17, represents most hospital outbreak and clinical isolates, apparently because it successfully adapted to hospital environments.

The >400 strains analyzed in this study were selected from a large representative collection of 2,000 *E. faecium* isolates. A wide variety of sources were used as selection criteria: hospital-associated outbreaks; clinical samples and stool samples from hospitalized patients, healthy persons, and animals; and a wide geographic distribution (21 countries on 5 continents).

Complex-17 probably evolved from the primary *E. faecium* ancestor ST-22 through a combination of mutation and recombination. The following observations suggest that recombination has been especially important in the genetic diversification of the *E. faecium* population: 1) within clonal complexes, most SLVs (73%) have arisen by recombination rather than point mutations; 2) no positive correlation exists between the degree of allelic diversity and the number of nucleotide differences in nonidentical alleles; and 3) most (60%) of the comparisons of MLST gene tree topologies were incongruent.

**Figure 4:**
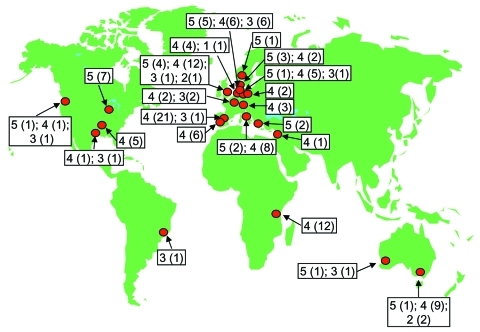
Global distribution of complex-17 isolates. Red circles indicate cities where complex-17 isolates were recovered. Numbers indicate epidemiologic sources: 1, animal isolates; 2, human community surveillance isolates; 3, surveillance (feces) isolates from hospitalized patients; 4, human clinical isolates; 5, isolates from documented hospital outbreaks. Numbers of isolates are indicated in parentheses.

Exploitation of a novel ecologic niche as hospital settings by *E. faecium* ST-17 often starts with adaptive changes (31). On the basis of our findings, we postulate that ST-17 acquired ampicillin resistance and a novel putative PAI. This amplifying selective process in which variants with a selective advantage can more easily acquire additional adaptive mechanisms has been called "genetic capitalism" (32). After successfully exploiting the hospital environment, ST-17 increased in frequency to become the dominant clone. Genetic diversification over time finally resulted in a meroclone, complex-17, of highly related genotypes, fully adapted as a nosocomial pathogen, that has spread globally (Figure 4). In addition, *E. faecium* STs, predominantly ST-78, belonging to complex-17, have recently also been found in the Republic of South Korea (K.S. Ko and J.-H. Song, pers. comm.). Considering the short period in which multiresistant *E. faecium* emerged as a nosocomial pathogen (33), complex-17 represents the first globally dispersed nosocomial-adapted clonal lineage. Despite the frequency of recombination events, clonal complex-17 is still detectable within the *E. faecium* population, which suggests that the emergence of this complex is relatively recent.

The existence of epidemic clones, even in recombining populations, is also seen in other bacterial species (34,35). However, the evolution of a single epidemic and clinically relevant genetic complex, as seen with *E. faecium*, differs from the evolution of other gram-positive pathogens like *Streptococcus pneumoniae* and *Staphylococcus aureus*. In *S. pneumoniae*, pandemic clones such as ST81, ST90, and ST156 represent major invasive and multidrug-resistant isolates that have spread globally (36). The allelic profiles of these clones, however, are highly diverse, which suggests that they are genetically unrelated and do not constitute a single genetic lineage, as does *E. faecium*. Furthermore, the serotype of *S. pneumoniae* seems a more important marker of invasiveness than the overall genotype (37). In *S. aureus* isolates, major pandemic MRSA clones that are responsible for most hospital-acquired infections are found in multiple genetically unrelated lineages, though most previously identified pandemic clones are found in clonal complex 8 (38,39). Therefore, the genetic diversity of major epidemic clones as seen in *S. pneumoniae* or *S. aureus* may not have yet emerged in *E. faecium* epidemic populations.

Stress-inducing conditions in hospitals, such as antimicrobial drug use, may have favored the selection of an enterococcal subpopulation, complex-17, with enhanced antibacterial resistance, virulence, and ability to spread. Whether reducing antimicrobial selection pressure in hospitals will reestablish a susceptible and less transmissible enterococcal population is unknown and will at least partly depend on the relative fitness costs of sustaining antimicrobial resistance and virulence determinants in *E. faecium*. Furthermore, the hospital-adapted complex-17 has rapidly spread globally during the last 2 decades. Subsequent acquisition of *vanA*- or *vanB*- containing transposons by horizontal gene transfer resulted in VRE with pandemic potential. Rapid diagnosis of complex-17 strains based on multiple locus variable number of tandem repeat analysis (MLVA) may help control its spread (40). Whether this effort will be successful depends on the level of complex-17 endemicity in the hospital. In many European countries, a relatively large community reservoir of VRE exists, a result of the massive use of the antimicrobial drug avoparcin as a growth promoter, while in general the prevalence of hospital-adapted (complex-17) VRE is much lower. In such a setting, hospital transmission of isolates belonging to complex-17 can be halted by using a fast genotyping scheme like MLVA to discriminate between hospital-adapted (complex-17) and community *E. faecium* strains followed by strict infection control measures. The combination of infection control measures plus genotyping controlled an outbreak of VRE in a Dutch hospital (41).

Establishing nosocomial co-endemicity of VRE and MRSA will facilitate the horizontal transfer of *vanA*- or *vanB*-containing transposons, transforming MRSA into VRSA, with implications for patient care. Until now, 3 sporadic cases of *vanA*-induced VRSA have been reported in the United States in 2002 and 2004. Spread of multidrug-resistant *E. faecium* strains and their resistance genes will have serious implications for health care, and control efforts should focus on early detection of *E. faecium* isolates belonging to complex-17.
